# Common spatial patterns of trees in various tropical forests: Small trees are associated with increased diversity at small spatial scales

**DOI:** 10.1002/ece3.7640

**Published:** 2021-05-27

**Authors:** Pavel Fibich, Vojtěch Novotný, Sisira Ediriweera, Savitri Gunatilleke, Nimal Gunatilleke, Kenneth Molem, George D. Weiblen, Jan Lepš

**Affiliations:** ^1^ Faculty of Science University of South Bohemia České Budějovice Czech Republic; ^2^ Biology Centre CAS Institute of Entomology České Budějovice Czech Republic; ^3^ Department of Science and Technology Uva Wellassa University Badulla Sri Lanka; ^4^ Department of Botany University of Peradeniya Peradeniya Sri Lanka; ^5^ New Guinea Binatang Research Center Madang Papua New Guinea; ^6^ Department of Plant Biology Bell Museum University of Minnesota St Paul MN USA

**Keywords:** individual species–area relationship, null model, spatial pattern, species diversity, species evenness, tropical forest

## Abstract

Tropical forests are notable for their high species diversity, even on small spatial scales, and right‐skewed species and size abundance distributions. The role of individual species as drivers of the spatial organization of diversity in these forests has been explained by several hypotheses and processes, for example, stochastic dilution, negative density dependence, or gap dynamics. These processes leave a signature in spatial distribution of small trees, particularly in the vicinity of large trees, likely having stronger effects on their neighbors. We are exploring species diversity patterns within the framework of various diversity‐generating hypotheses using individual species–area relationships. We used the data from three tropical forest plots (Wanang—Papua New Guinea, Barro Colorado Island—Panama, and Sinharaja—Sri Lanka) and included also the saplings (DBH ≥ 1 cm). Resulting cross‐size patterns of species richness and evenness reflect the dynamics of saplings affected by the distribution of large trees. When all individuals with DBH ≥1 cm are included, ~50% of all tree species from the 25‐ or 50‐ha plot can be found within 35 m radius of an individual tree. For all trees, 72%–78% of species were identified as species richness accumulators, having more species present in their surroundings than expected by null models. This pattern was driven by small trees as the analysis of DBH >10 cm trees showed much lower proportion of accumulators, 14%–65% of species identified as richness repellers and had low richness of surrounding small trees. Only 11%–26% of species had lower species evenness than was expected by null models. High proportions of species richness accumulators were probably due to gap dynamics and support Janzen–Connell hypothesis driven by competition or top‐down control by pathogens and herbivores. Observed species diversity patterns show the importance of including small tree size classes in analyses of the spatial organization of diversity.

## Graphical abstract

Common spatial patterns of trees were observed in three tropical forest plots. Small trees were associated with increased diversity, while large trees were more often associated with low diversity. Gap dynamics and negative density‐dependent processes were expected to form the diversity patterns.

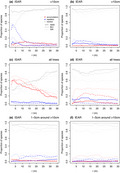

## 1 INTRODUCTION

Explaining the spatial distribution of diversity in tropical forests, reaching high values already at small spatial scales, is a conundrum that has been examined by multiple studies (Chesson, 2000; Hubbell, 2006; Janzen, 1970; Wills et al., 1997). Commonly observed clumped (aggregated) spatial distribution of individual species leads to increased intraspecific interactions between neighbors in close proximity (Condit et al., 2000; Law et al., 2009), although in mature tropical forests, the species identities of nearest neighbors often correspond to their random mixing (Lieberman & Lieberman, 2007). For example, in Barro Colorado Island tropical forest plot, 20 nearest neighbors comprise on average 14 species (Hubbell, 2006). The stochastic dilution hypothesis (Wiegand et al., 2012) predicts that with increasing species richness, stochastic effects overpower signals of spatial associations between species. The predictability of neighboring species identity was therefore observed to decrease with increasing species richness in various ecosystems (Chacón‐Labella et al., 2017; Wang et al., 2016; Wiegand et al., 2012).

Multiple spatial scales and various tree size classes included in the analyses may help to disentangle the effects of past and ongoing processes maintaining diversity in forest communities. The framework based on individual species–area relationship (ISAR) allows to isolate the role of individual species in structuring spatial diversity of the vegetation (Wiegand, Gunatilleke, Gunatilleke, & Huth, 2007) and examines the diversity on different spatial scales (spatial grains, that is, different distances from the focal individual trees). ISAR allows to classify species into “richness accumulators,” “richness repellers,” and richness neutral species if the trees of these species are surrounded by more, fewer, or expected number of species compared with some null model. Richness accumulators may be facilitated by positive species‐to‐species interactions, stronger intra‐ than interspecific competition, or top‐down, density‐dependent control by pathogens or herbivores, while repellers indicate strong interspecific competition (Espinosa et al., 2015; Wiegand, Gunatilleke, Gunatilleke, & Huth, 2007).

Although the majority of individuals of trees in tropical forests belong to small‐size classes (e.g., 91% of trees in Wanang plot, Papua New Guinea, and 84% of trees in Barro Colorado Island plot, Panama, both 50 ha, have DBH <10 cm), most of the studies using ISAR in tropical forests are based on large trees only (commonly trees with DBH >10 or >20 cm; Wiegand, Gunatilleke, Gunatilleke, & Huth, 2007, but see Yang et al., 2013). This is because only the large trees are expected to affect the surrounding diversity. On the other hand, including small trees will add new small‐size species that may have different coexistence strategies (Aarssen et al., 2006), and some small trees may have limited dispersal or specific habitat preferences. For example, newly appeared gaps can host several species of small trees. Thus, including small trees in the diversity analysis can provide a more complete view of the forest dynamics and structure, reflecting also better the state of vegetation before self‐thinning of small trees.

Highly skewed species abundance distribution in the tropical forests suggests that species richness alone cannot fully describe species diversity (Kirwan et al., 2007; Zhang et al., 2012). Evenness (Pielou, 1969) is a suitable descriptor of the relative frequency of interspecific and intraspecific interactions within communities (Hillebrand et al., 2008), although many other factors may affect local variation of species abundances (e.g., dispersal limitation, habitat heterogeneity). We therefore designed a new spatially explicit evenness function, similar to ISAR and spatially explicit Simpson index (Shimatani & Kubota, 2004; Wiegand & Moloney, 2014), which allows to quantify the evenness of the vegetation in the neighborhood of individual focal trees.

We have analyzed spatial diversity patterns for individual species to study whether including small tree sizes change species diversity patterns, using data from three tropical forests: Wanang plot in Papua New Guinea with ~580 tree species, Barro Colorado Island in Panama with ~300 tree species, and Sinharaja plot in Sri Lanka with ~240 tree species. In particular, we explore the following four hypotheses: (1) The proportion of neutral species increases with increasing species richness. In species‐rich forest plots, vegetation dynamics is expected to be dominated by stochastic effects, even if the community is structured by deterministic niche processes (McGill, 2010; Wiegand et al., 2012), making the detection of consistent patterns of interactions with neighbors difficult (Wang et al., 2016; Rajala et al., 2018, but see Wiegand, Gunatilleke, & Gunatilleke, 2007). Following stochastic dilution hypothesis (Wiegand et al., 2012), we expect the importance of interspecific interactions and therefore the proportion of non‐neutral species (accumulator and repeller species) to decrease with increasing species richness in the plots (although other factors such as dispersal and habitat heterogeneity should be considered too). (2) Including small trees increases the strength of nonrandom species diversity patterns. In comparison with other studies that have considered only large trees, by including small trees in the analyses we expect to observe stronger diversity patterns than those based on only large trees, for example, increased presence of diversity repellers due to the space occupied (limited) by dense population of their offspring around adult trees, or increased number of diversity accumulators due to gap dynamics as gaps offer opportunity for small trees from many species; Lieberman et al., 1989). On the contrary, including several size classes may blur diversity patterns, for example, because habitat preferences are changing during the ontogeny of trees (Comita et al., 2007; Punchi‐Manage et al., 2013). Small trees also suffer the highest mortality and the strongest competition (Comita & Hubbell, 2009). For example, Janzen–Connell hypothesis suggests that conspecific offspring suffers higher mortality than heterospecific individuals around large trees due to stronger conspecific negative density dependence caused by specialized pathogens and herbivores infecting small trees from the large mother ones (Janzen, 1970). (3) Large focal trees are surrounded by low species diversity of small trees. Because of commonly observed negative effects of large trees on surrounding small trees (Punchi‐Manage et al., 2015), we similarly expect negative effects of large trees on the diversity of small trees in their close vicinity, mostly due to asymmetric competition, although stronger intraspecific than interspecific competition (Comita et al., 2007) and dispersal limitation or habitat preferences may change diversity patterns. (4) Evenness patterns are more often neutral than the species richness patterns. The low effect of species dominance is expected due to the species dilution hypothesis in species‐rich forests. By computing the spatial variation of evenness, we expect to identify species with strong dominance effects in their surroundings that may not be captured by ISAR.

Our study is the first showing species spatial diversity associations in the tropical forest plot with >500 tree species (being far outside of already studied number of species range) with considering all trees (but see Yang et al., 2013 based on Barro Colorado Island plot) and applying spatially explicit evenness in the analyses.

## 2 METHODS

### 2.1 Study sites

We tested diversity associations in three tropical forest plots (Table S1) that are part of the ForestGEO network (Anderson‐Teixeira et al., 2015): Wanang plot, Papua New Guinea (hereafter abbreviated as WAN), Barro Colorado Island, Panama (BCI), and Sinharaja, Sri Lanka (SIN). In each plot, every stem ≥1 cm diameter at breast height (DBH) was tagged, mapped (with *x* and *y* coordinates), and identified to species and its DBH was measured, using standardized protocols. Climbers were excluded. WAN is a plot in the lowland tropical wet mixed evergreen forest and was established in 2009. BCI is a plot in the seasonally moist tropical forest and established in 1981. SIN is a plot in the wet tropical forest and was established in 1993. Plot size, environmental characteristics, and the number of stems and species are presented in Table S1, and more details and references are in Vincent et al. (2015) and Vincent (2015) for WAN, in Condit (1998), Condit et al. (2000) and Condit et al. (2019) for BCI, and in Punchi‐Manage et al. (2013, 2015) for SIN.

### 2.2 Spatial richness and evenness association functions

We applied an individual species–area relationship (ISAR) that quantifies the spatial structure of the local species richness around individuals of a focal species (Wiegand, Gunatilleke, Gunatilleke, & Huth, 2007; Wiegand & Moloney, 2014). ISAR is commonly used spatial pattern analysis method combining Ripley's *K* function and species–area relationship function, as it counts the number of species around the mean individual of a particular species with increasing distance. We calculated ISAR as

$${\text{ISAR}}_{i}\left(r\right)=\sum_{j=0}^{S}P_{\mathrm{ij}}\left(r\right)$$

where ISAR*_i_*(*r*) is the individual species–area relationship for species *i* at distance *r*; *S* is the number of species; and *P_ij_*(*r*) is the proportion of the individuals of species *i* (focal species) that have at least one individual of species *j* within distance *r*. Moreover, we used species evenness as another diversity characteristic considering relative abundance of species at a particular radius around the focal species (for hypothesis 4). As an analogy to ISAR, we designed IEAR, or individual species area evenness relationship. Evenness for the IEAR was calculated as

$${\text{IEAR}}_{i}\left(r\right)=\frac{‐\sum_{j=0}^{S}P_{\mathrm{ij}}\left(r\right)*\ln\left(P_{\mathrm{ij}}\left(r\right)\right)}{\ln(S)}$$

that is the Shannon diversity index divided by its maximum value when all the species present are represented by the same number of individuals, which is ln(*S*) in our case (Pielou, 1969) for the set of trees within the particular radius around the focal tree, and then averaged for all trees from the focal species. IEAR quantifies how even are abundances of species distributed around particular focal species across different spatial distances. For example, the most even species distribution, when all the species are represented by the same number of individuals, has value 1, while low values of evenness close to 0 would be expected for a community with strong dominance of a single species. Evenness is an aspect of diversity complementary to the number of species—unlike the Simpson index (Shimatani & Kubota, 2004) and the Shannon index (Reardon & O'Sullivan, 2004), it is not be mathematically related to the number of species. We decided to use the evenness based on the Shannon (instead of Simpson) index, because the former is more sensitive to the relative representation of rare species.

The ISAR and IEAR were calculated for trees from the following size categories (for hypothesis 2): (1) all trees (DBH ≥1 cm), (2) trees with DBH >5 cm, and (3) large trees with DBH >10 cm. We also calculated between size classes (cross) versions of ISAR and IEAR functions for trees in 1–5 cm DBH range around the focal species with DBH >10 cm to study the species richness and evenness associations of small trees around large trees (for hypothesis 3, as in Espinosa et al., 2015). In all four analyses, the focal species had to have ≥50 individuals in the size class examined. All other species, no matter how abundant, were used as the target species, analyzing their species richness and evenness around the focal trees.

### 2.3 Null models and ecological interpretations

The observed species diversity patterns were compared with null models (Wiegand & Moloney, 2014). We applied inhomogeneous (heterogeneous) Poisson null model (INM) where the positions of all trees of nonfocal species remain fixed and the new positions of only focal species trees were simulated. In INM, the spatially explicit density of individuals of the focal species is first nonparametrically estimated as a function of coordinates within the plot using a Gaussian kernel with *SD* (bandwidth) of 35 m (similar *SD* was used in smaller 1‐ha plot in PNG by Fibich et al., 2016; Das et al., 2018 tested various *SD*s and found just few small differences between results). Then, the positions of individual trees are randomly generated according to the density function estimated in the previous step. Reflecting density of individuals accounts for possible heterogeneous density of individuals over the plot that may be caused by environmental gradients that are often considered the most likely causes of broad‐scale patterns, although a dispersal gradient cannot be excluded either (Wiegand & Moloney, 2014). By not considering varying density of individuals, the diversity neutral species would be seen as diversity attractor, if its preferred habitat is relatively species‐rich, and as diversity repeller, if the preferred habitat is species‐poor.

The significance of the deviation of individual diversity functions from the null model was tested by 199 independent null model realizations and one global goodness‐of‐fit test (Law et al., 2009; Loosmore & Ford, 2006). To avoid the problem of simultaneous inferences of pointwise envelopes, we used maximum absolute deviation (MAD) test for each focal species for the whole 0–35 m spatial distance range (Chanthorn et al., 2018; Myllymäki et al., 2017; Wiegand & Moloney, 2014). In the first step, standardized effect sizes (SES) applied on simulated ISAR functions transform envelopes to have a constant width, and then, scale‐independent global envelopes limits were computed. The deviations of observed functions from the null model (SES of observed patterns above/below the global envelope limits) can be visualized along spatial distances (Chanthorn et al., 2018; Wiegand, Gunatilleke, Gunatilleke, & Huth, 2007).

The species with higher ISAR or IEAR values compared with the null model by goodness‐of‐fit test are considered richness (ISAR) or evenness (IEAR) accumulators, as they are associated with higher than expected number of species or species evenness. If the observed values are lower than those from the null models, the species are considered richness repellers or evenness repellers. The values not different from the model's simulations indicate that species behave as richness neutral or evenness neutral. The non‐neutral effect of individual species on local diversity (richness or evenness) might be result of various processes: Either (1) the focal individual influences its neighbor, or (2) the neighbors influence the (establishment, growth, and survival of) focal individual, or (3) both focal individual and surrounding are affected by its (biotic or abiotic) surrounding. (1) The focal individual can increase the number of conspecific individuals simply by dispersing its seeds in its vicinity; in this way, it would also increase the competitive pressure on other species, and thus could decrease their number (i.e., species richness), causing a species to be a richness repeller. It is, however, not clear how this might affect the evenness, but because of differences in sensitivity to competition, we can expect also decreased evenness. On the contrary, if we consider the Janzen–Connell effects, focal species’ own offspring might be affected by species‐specific enemies, and thus provide space for more heterospecific individuals, and thus more species, abundances of which, however, need not be more or less evenly distributed. All these effects can be expected mainly for larger focal trees and their smaller neighbors (hypotheses 3). (2) According to the safety in diversity hypothesis (Wills et al., 1997), the spots with high species richness are safer from enemies and thus more suitable for establishment. If this is so, the highly diverse spots should be detectable when all the individuals are included in the analysis (the spots or gaps may contain more size classes of trees, hypotheses 2; Lieberman et al., 1989). According to this hypothesis, not only the number of species but also their evenness should play a role (hypothesis 4). (3) The local diversity (both number of species and evenness) is affected by the surroundings—it might be topography or environmental conditions, but in tropical forest very probably the biotic surrounding. Typically, the gap phase provides more light due to biomass removal by disturbance (Clark & Clark, 1992) and thus space for many small trees of higher number of species, but this may not increase the evenness. These effects should again be stronger if all the individuals are taken into account.

Only trees located at a distance ≥*r* from the border of the plot were considered when computing ISAR(*r*) and IEAR(*r*) functions to avoid the edge effect. Spatial patterns for individual species are described in the Supplementary materials B. We used R version 3.5.1 (R Core Team, 2018) with packages “spatstat” version 1.56‐1 (Baddeley et al., 2015) for spatial pattern analyses, “vegan” version 2.5‐2 for diversity indices (Oksanen et al., 2017), and modified R code for ISAR available in Fibich et al. (2016).

## 3 RESULTS

**FIGURE 1:**
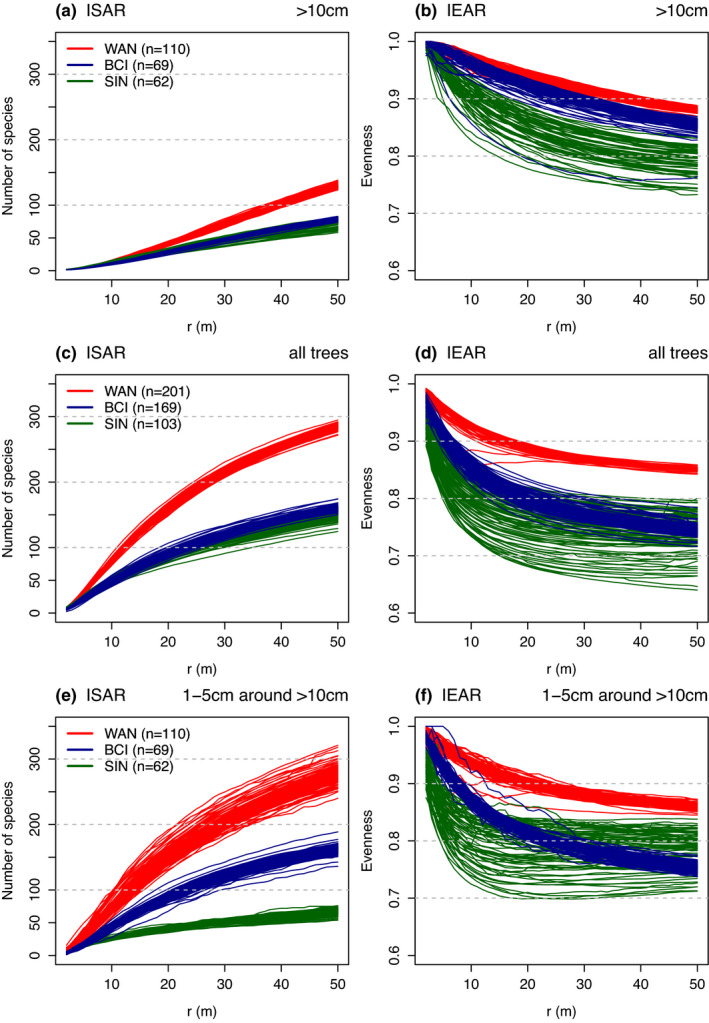
Observed individual species diversity ISAR (species richness) and IEAR (evenness) curves for WAN, BCI, and SIN tropical forest plots and trees with DBH >10 cm, all trees (≥1 cm), and 1–5 cm DBH trees around >10 cm DBH trees within the increasing radius *r* (m). *n* corresponds to the number of focal species, each shown as a single curve

Visually, there is a gradient from relatively constant number of species and evenness in 50 m × 50 m spatial grid cells in the WAN plot, through more variable diversity in the BCI plot to SIN plot with the highest variability in the number of species and evenness (Figures S1 and S2). The number of species recorded within 35 m radius (i.e., 3,848 m^2^) from the focal tree represented a high proportion of the total tree diversity found in 25 ha, for instance, ~220–250 species from the total of 522 in WAN, ~120–150 species from 272 in BCI, and ~100–140 species from 236 in SIN (Figure 1). Variability of ISAR and IEAR among species seems to be, respectively, smaller and higher in SIN than the other plots.

For all trees (DBH ≥1 cm), there were higher proportions of non‐neutral species (for both richness and evenness) than if the analysis was limited to large trees with DBH >5 or >10 cm (Table 1). There were more accumulators than repellers of species richness and vice versa for evenness. Overall increase in proportion of neutral species with increasing plot species richness was observed for evenness and only for between tree size association of species richness (hypothesis 1, Table 1). On the other hand, proportion of non‐neutral species decreases with increasing size of included trees (hypothesis 2, Table 1).

**TABLE 1:** Percentages of significant (*p* < .05) accumulator/repeller species under inhomogeneous null model (INM) for ISAR and IEAR based on MAD goodness‐of‐fit test (Wiegand & Moloney, 2014) on 0–35 m distance for trees with >10 cm DBH, >5 cm DBH, all trees (DBH ≥1 cm), and 1–5 cm DBH trees around >10cm DBH trees

Plot	No spp.	>10 cm DBH			>5 cm DBH			All trees			1–5 cm around >10 cm DBH	
		*n*	ISAR %	IEAR %	*n*	ISAR %	IEAR %	*n*	ISAR %	IEAR %	ISAR %	IEAR %
WAN	581	110	21/14	1/18	199	42/10	12/16	201	73/11	9/25	9/11	2/3
BCI	302	69	7/65	3/22	120	27/44	13/16	167	72/7	35/11	3/20	5/16
SIN	236	62	29/29	11/26	88	49/23	27/23	103	78/5	46/11	27/31	23/14

Note

*n* is the number of analyzed focal species (*n* for 1–5 cm around >10 cm is the same as for >10 cm).

**FIGURE 2:**
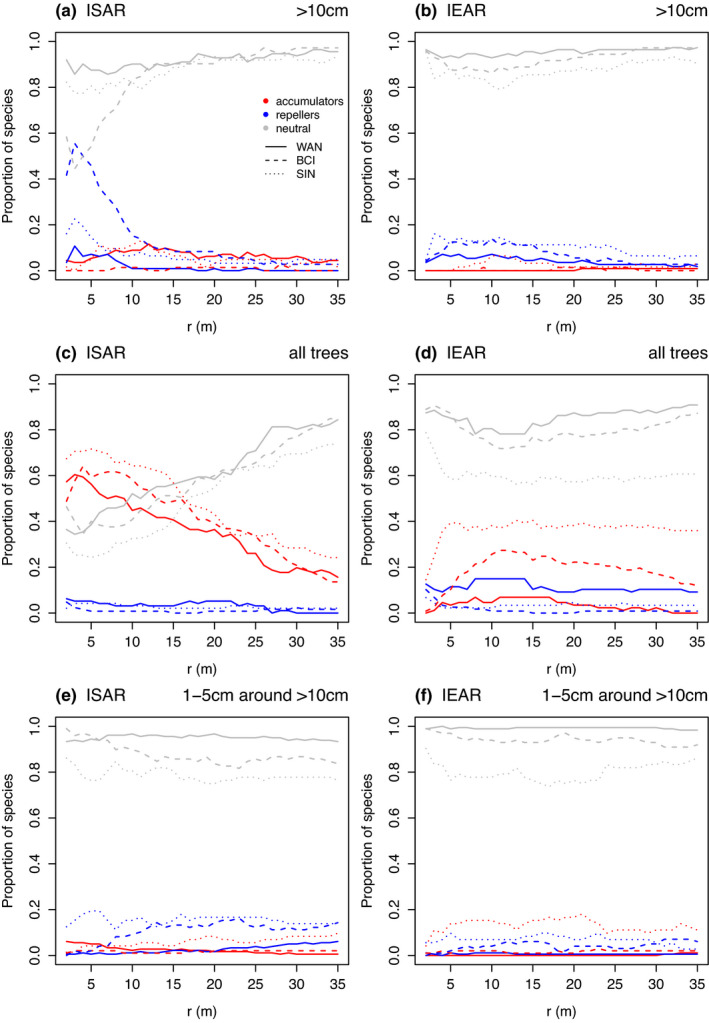
Proportions of individual species showing more (i.e., accumulator), less (i.e., repeller), and expected (i.e., neutral) richness (ISAR) and evenness (IEAR) associations for trees with DBH >10 cm, all trees (≥1 cm), and 1–5 cm DBH trees around >10 cm DBH trees in WAN, BCI, and SIN tropical forest plots with increasing spatial distance (radius *r* (m)). Species were classified by MAD test (Wiegand & Moloney, 2014) where observed values were higher than global envelope limits of inhomogeneous null model for accumulators, lower for repellers, or within the null model for neutral species

The highest proportion of non‐neutral species for both richness and evenness was found on the spatial distances between 0 and 15 m (Figure 2). For large trees (>10 cm DBH), there was a peak in the proportion of richness repellers at the ~3 m distance consistent in all three plots (Figure 2a). Richness accumulators formed a small peak, comprising ~20% species, at 12 m distance in WAN and SIN, while at BCI never represented more than 5% of species at all spatial distances. IEAR showed a relatively stable proportions, <15% of evenness repellers and <10% of evenness accumulators, across all spatial distances (Figure 2b).

For all trees (DBH ≥1 cm), the ISAR analysis showed almost complete absence of richness repellers, while the richness accumulators peaked at the ~5–10 m spatial distance, comprising ~60%–70% of all species, and monotonously decreasing from there with increasing distance. In contrast, the IEAR patterns differed between plots (Figure 2d). While there was a uniform proportion of evenness repellers, at ~15% of species in WAN, there were few repellers at the other two plots. Further, BCI and SIN had a broad peaks of evenness accumulators (maximum 40% and 30% of all species in SIN and BCI) at >10 m distance, while in WAN exhibited a slow monotonous decrease in the accumulators from the maximum at 10–15 m distance (up to 10% of all species at WAN).

For trees with DBH >5 cm, there were more accumulators and less‐intensive repellers peak similar to >10 cm DBH trees in all plots for ISAR and IEAR (Figure S3).

### 3.1 Diversity of small trees around large trees

Richness and evenness associations of trees with 1–5 cm DBH around trees with DBH >10 cm were weak, mostly below 20% of all species (hypothesis 3, Figure 2e,f). The trends differed among the plots: Richness repellers peaked at 20% of all species at 5 m distance in SIN, increased steadily from 0% to 20% over 0–23 m distances in BCI, and remained below 10% of species WAN. The richness accumulators represented at most 10% of species in SIN, and mostly, none were observed in other two plots. Evenness repellers were mostly below 10% of species in all plots, and only SIN had up to 20% of evenness accumulators (Figure 2e).

## 4 DISCUSSION

Wanang (WAN), Barro Colorado Island (BCI), and Sinharaja (SIN) are tropical forest plots quite different from each other in terms of species richness, biogeographic location, climate seasonality, or environmental heterogeneity. Despite these differences, we found strong common patterns for all of them, namely (1) peaks of species richness repellers for short distances when analyzing large trees (DBH >10 cm), (2) peaks of species richness accumulators, representing over 70% of all tree species, at short distances when considering all trees (DBH ≥1 cm) in all plots, (3) species richness accumulators being mostly more frequent than repellers along 0–35 m spatial distance, except between size class analyses, where for most species of large trees were repellers for the richness of small trees, and (4) weak dominance effect reflected by mostly weak patterns for IEAR (only up to 26% species had lower species evenness than was expected by the chance). Species richness accumulator peak on small spatial distances is similar to species‐rich shrubland (Chacón‐Labella et al., 2016; Perry et al., 2017)and suggests a common behavior of species‐rich ecosystems.

The majority of individual trees in tropical forests are small. We have shown that analyzing only large (e.g., >10 cm DBH) trees or trees from limited size ranges leads to results different from those obtained using trees of all size classes. For example, ISAR curves reached values approximately two times higher for all individuals compared to trees with DBH >10 cm. Peaks of non‐neutral spatially explicit richness and evenness were mostly within 15–20 m radius from the focal trees, indicating the spatial distance of interactions between neighboring trees (Law et al., 2009). Generally, non‐neutral richness and evenness associations are ascribed to (1) between‐species interactions (competition leads to repellers), (2) habitat associations (favorable sites with high diversity patches lead to accumulators), and (3) spatial patterns and local dominance (e.g., more abundant species have higher probability to be classified as accumulators than less abundant species; Punchi‐Manage et al., 2015, but see Chacón‐Labella et al., 2017 where they have shown that highly abundant species are not necessarily diversity non‐neutral species). The fact that the analysis of the effect of large on small individuals yielded more repellers than the other analyses suggests that the positive associations are mainly caused by the response of establishing individuals to the habitat preferences, supporting thus either the safety in diversity hypothesis (Wills et al., 1997), or the effect of gaps that are generally species‐rich (particularly when individuals of all sizes are included) and, at the same time, have higher chance for the establishment of new individuals.

The expected increase in the proportion of neutral species diversity associations with increasing number of species (hypothesis 1) in the plots was confirmed just for evenness (dominance), not for species number (Table 1). For such species‐rich sites, other individual site differences may have stronger effects on diversity associations than species number. For example, the higher proportion of richness accumulators in WAN compared with BCI can be also due to differences in the environmental heterogeneity (that may be reflected by stronger individual species spatial aggregation in WAN than in BCI, even after accounting for inhomogeneous density of individuals, Supplementary materials B). Moreover, there was higher proportion of small trees in WAN than in BCI (91% vs. 84% of trees with DBH <10 cm in WAN and BCI, respectively). Small trees are more often clumped due to stronger effect of dispersal processes than large trees that are under self‐thinning competition for longer time and have stronger habitat preferences (Comita et al., 2007; Fibich et al., 2016).

### 4.1 Effects of tree sizes

Small tree spatial patterns are often different from the large individuals, for example, due to different habitat associations; moreover, small‐size species may have also different dispersal strategies that both may contribute to species coexistence (Aarssen et al., 2006). Even though large trees (DBH > 10 cm) constitute approximately half of the mature forest biomass, they represent only a fraction of overall forest species richness (Lutz et al., 2018). The majority of trees in tropical and other forests belong to the smallest DBH ranges; therefore, the analysis limited to large individuals or DBH ranges provides only a partial picture of the forest community. We observed contrasting spatially explicit species richness associations between all trees and the subset of large trees. For example, the large trees (>10 cm DBH) in WAN reached on average 140 target species, compared with 300 target species for all trees (DBH ≥ 1 cm) when using ISAR curves for 50 m radius. The ISAR analysis of all trees provided a peak of species richness accumulators consistent across the sites. This contrasts with the analysis restricted to large trees (>10 cm DBH), where only few richness accumulators appear, together with the peak of richness repellers at the short spatial distance. Similarly, ISAR analyses in SIN showed a peak in richness accumulators for small trees (only 1–5 cm DBH trees) at the smallest spatial distances, but an increasing proportion of repellers with increasing focal trees sizes (Punchi‐Manage et al., 2015).

We expect that the peaks of species richness accumulators among all trees might be generated by gap (phase) dynamics that is a dominant process in the tropical forest turnover (Gray & Spies, 1997; Hubbell et al., 1999; Kohyama, 1993; Punchi‐Manage et al., 2015; Velázquez & Wiegand, 2020). Gaps are caused by tree or branch falls, less frequently by other disturbances such as landslides. They provide favorable conditions, including low competition, for establishment and survival of trees (Clark & Clark, 1992). Later, in the gaps with many small trees, clumps of conspecifics are strongly reduced by the effects of negative density‐dependent processes (e.g., natural enemies and stronger intra‐ than interspecific competition, Janzen, 1970; LaManna et al., 2017) and therefore can provide space for other species. Gaps are usually more species‐rich than the surrounding nongap vegetation because they comprise higher density of mostly small trees compared with the surrounding understory (Clark & Clark, 1992). Density‐dependent processes lead to stronger reduction in conspecific than heterospecific neighbors, and their effects are often more pronounced for smaller trees in gaps than for larger trees in the surrounding nongap vegetation (Comita & Hubbell, 2009). Higher seedling establishment and higher numbers of species were indeed found in the gaps than nongap sites in BCI (Hubbell et al., 1999). More abundant species may have higher chance to be classified as diversity non‐neutral species, because their diversity associations are more likely to be recognized by statistical tests in the noise of large neighborhood variability (Punchi‐Manage et al., 2015; Wang et al., 2016). This is demonstrated also by our ISAR analysis in all three plots (Figure 2), where decreasing DBH threshold increases the number of included trees and, as a consequence, decreases the percentage of richness neutral species (hypothesis 2). Even though the high diversity gaps are mostly dominated by small trees, similar ISAR analysis based only on trees 1–5 cm DBH did not observe high peak of accumulators in SIN (Punchi‐Manage et al., 2015). It suggests that gaps or ancient gaps contain also some bigger trees too (Figure 2c). Based on nearest neighbor analysis, interspecific interactions are expected to be low in such species‐rich forests (Lieberman & Lieberman, 2007); therefore, positive between‐species interactions may be less likely to affect species richness associations (Wiegand, Gunatilleke, Gunatilleke, & Huth, 2007, Wiegand et al., 2012, but see Chacón‐Labella et al., 2017).

Spatial distance of species richness repeller peaks was related to the average area occupied by the focal tree (Wiegand, Gunatilleke, Gunatilleke, & Huth, 2007). It is difficult for other trees, particularly larger trees, to grow in close vicinity of the focal tree. Average area occupied by a tree with DBH >10 cm was 24.5 m^2^ in BCI, 19.5 m^2^ in WAN, and 14.7 m^2^ in SIN. This can explain observed peaks of richness repellers at the 5–10 m radius distances, and repellers are thus related to competition (Espinosa et al., 2015). Moreover, although we applied inhomogeneous null model that partially filters out large‐scale density gradient (bandwidth 35 m), generally, strong differential responses of individual species to small‐scale habitat heterogeneity may explain the presence of repellers (Das et al., 2018; Plotkin et al., 2002).

### 4.2 Diversity of small trees around large trees

Regardless of other biotic interactions, trees tend to generate aggregated conspecific clusters by limited dispersal or by response to environmental heterogeneity. At the same time, large trees might promote high species diversity in their immediate vicinity. For example, Janzen–Connell hypothesis suggests that distance‐ or density‐dependent mortality of seedlings and saplings resulting from infection by specialized pathogens or herbivores from the parent tree is stronger for conspecifics than for heterospecifics (Janzen, 1970). Accordingly, we observed more species richness repellers than accumulators for small trees around large trees (as expected in hypothesis 3). Close to the mother tree, conspecifics were outcompeted and further away there was lower conspecific negative density and distance effect (Murphy et al., 2017); therefore, conspecific could survive there more easily. Finally, during the growth of tree we expect decreasing species richness in the surrounding vegetation because long competition with neighbors reduces their numbers.

Overall in all plots, the majority of species has the expected diversity of small trees around large trees. This corresponds to the conclusions that the effects of large trees on diversity of small trees were mostly weak (Punchi‐Manage et al., 2015). Such results strongly support stochastic dilution hypothesis (McGill, 2010; Wiegand et al., 2012).

### 4.3 Spatially explicit evenness

Species evenness captures the relative strength of interspecific and intraspecific interactions within communities (Kirwan et al., 2007). IEAR classified higher proportions of species as evenness repellers than accumulators, although the majority of species were evenness neutral according to IEAR (in concordant with hypothesis 4). Evenness repellers were not prevalent at any particular distance (maximally 26% of species were repellers in BCI), and their peaks correspond to the peaks of species richness repellers.

Weak spatial dominance effects support the stochastic dilution hypothesis (Wiegand et al., 2012), postulating that in species‐rich communities the spatial distribution of species is independent of one another and stochasticity may blur underlying deterministic niche processes structuring the community (Wang et al., 2016). The Janzen–Connell hypothesis suggests that density‐dependent mortality should increase the evenness and space availability in the tree community, and we therefore expect that new species can appear in such places. The observed trends do not match the expectation precisely, although there is a general tendency in both measures to decrease with the spatial distance. The evenness repellers, decreasing evenness in their neighborhood, could be tree species adapted to unusual habitats dominated by only a few other species sharing their habitat preferences. Such species were rare in our plots.

## 5 CONCLUSIONS

The majority of trees in the tropical forest belongs to small‐size classes that also include a majority of the local species pool. By using spatially explicit methods, we observed that many tree species behave as species richness accumulators and evenness neutral particularly when the vegetation in their closest neighborhood up to 35 m radius is considered. These patterns were observed for all trees (DBH ≥1 cm), although separate analysis of large trees showed mostly species richness neutral species. Whereas this pattern might be partially consequence of increasing power of the test with increased number of individuals, we expect that it is driven mainly by gap dynamics and negative density dependence processes (competition or top‐down control by pathogens and herbivores) that are more affecting small trees than large trees. Both processes support safety in diversity and Janzen–Connell hypotheses, although for the results based just on large trees stochastic dilution hypothesis is more appropriate.

## CONFLICT OF INTEREST

The authors declare that they have no conflict of interest.

## AUTHOR CONTRIBUTION

**Pavel Fibich:** Conceptualization (lead); Data curation (equal); Formal analysis (lead); Investigation (equal); Methodology (equal); Software (equal); Validation (equal); Visualization (equal); Writing‐original draft (equal); Writing‐review & editing (equal). **Vojtěch Novotny:** Conceptualization (equal); Data curation (equal); Funding acquisition (equal); Investigation (equal); Methodology (equal); Project administration (equal); Resources (equal); Supervision (equal); Validation (equal); Writing‐original draft (equal); Writing‐review & editing (equal). **Sisira Ediriweera:** Conceptualization (equal); Funding acquisition (equal); Investigation (equal); Resources (equal); Supervision (equal); Writing‐original draft (equal); Writing‐review & editing (equal). **Savitri Gunatilleke:** Conceptualization (equal); Funding acquisition (equal); Supervision (equal); Writing‐original draft (equal); Writing‐review & editing (equal). **Nimal** **Gunatilleke:** Conceptualization (equal); Funding acquisition (equal); Resources (equal); Supervision (equal); Writing‐original draft (equal); Writing‐review & editing (equal). **Kenneth Molem:** Data curation (equal); Methodology (equal); Validation (equal); Writing‐original draft (equal); Writing‐review & editing (equal). **George D. Weiblen:** Conceptualization (equal); Funding acquisition (equal); Resources (equal); Writing‐original draft (equal); Writing‐review & editing (equal). **Jan Lepš:** Conceptualization (equal); Data curation (equal); Formal analysis (equal); Investigation (equal); Methodology (equal); Software (equal); Supervision (equal); Validation (equal); Writing‐original draft (equal); Writing‐review & editing (equal).

## Supporting information

Supplementary Material

Supplementary Material

## Data Availability

All data are available through ForestGEO data request portal http://ctfs.si.edu/datarequest/ and BCI data also in Condit et al. (2019), Dryad https://doi.org/10.15146/5xcp‐0d46.
