# Supplementary material for: Common spatial patterns of trees in various tropical forests: Small trees are associated with increased diversity at small spatial scales
Source: Ecol Evol. 2021 May 27;11(12):8085–95. doi: 10.1002/ece3.7640 (PMC8216943; doi:10.1002/ece3.7640)
Supplement: Supplementary file 1 — Supplementary Material [file ECE3-11-8085-s002.pdf]

A. Supplementary materials of "Common spatial patterns of trees in various tropical forests: small trees are associated with increased diversity at small spatial scales" by Fibich P, Novotný V, Ediriweera S, Gunatilleke S, Gunatilleke N, Molem K, Weiblen GD, Lepš J.

Table S1. Plot characteristics for Wanang (WAN), Barro Colorado Island (BCI) and Sinharaja (SIN). Plot size in ha, Elevation range in m, MAT – mean average temperature in °C, MAP – mean annual precipitation in mm; Census – the census used for analysis; stems are characterized by the total number and % of stems with <10 cm DBH; species are characterized by the total number, the rarefied number per 25 ha area, the number of common species represented by ≥50 stems, and the number of common large species, represented by >50 stems with >10 cm DBH. Number of species was rarefied to 25 ha (size of the smallest plot in SIN) by calculating mean of 100 randomly located 25 ha squares in each 50 ha plots (500 m x 500 m squares were randomly shifted along the longest edge of the 50 ha plots). Observed, not rarefied, number of species is used in the text.

Plot	Size	Elevation Range	MAT	MAP	Census	Stems		Number of species			
						All	<10 cm DBH [%]	All	25 ha	≥ 50 Stems	≥ 50 Stems & >10 cm DBH
WAN	50	90-190	25.8	4000	1	288204	91	581	522	328	112
BCI	50	120-155	27.1	2600	7	221758	84	302	272	191	72
SIN	25	424-575	22.5	5016	1	204973	92	236	236	149	60

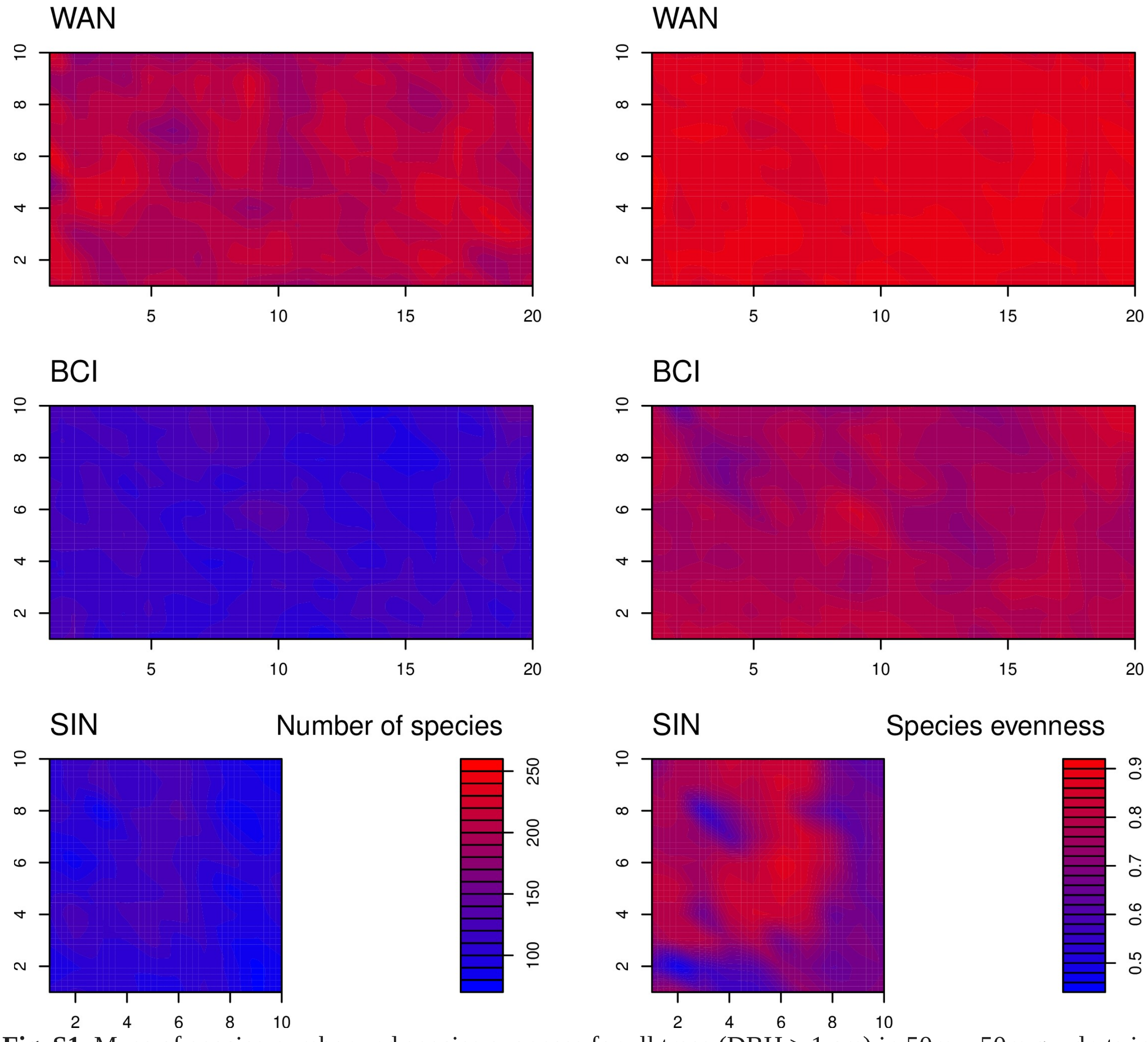

Fig. S1. Maps of species number and species evenness for all trees (DBH ≥ 1 cm) in 50m x 50m quadrats in Wanang (WAN), Barro Colorado Island (BCI) and Sinharaja (SIN) plots.

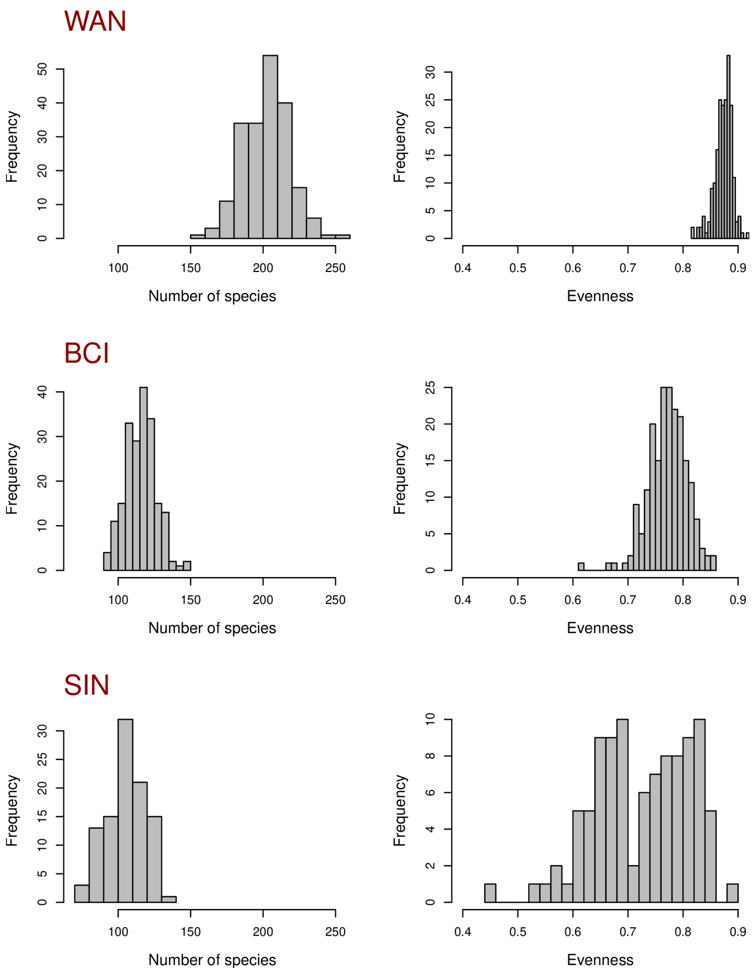

Fig. S2. Histograms of of species number and species evenness for all trees (DBH≥1 cm) in 50m x 50m quadrats in WAN, BCI and SIN plots.

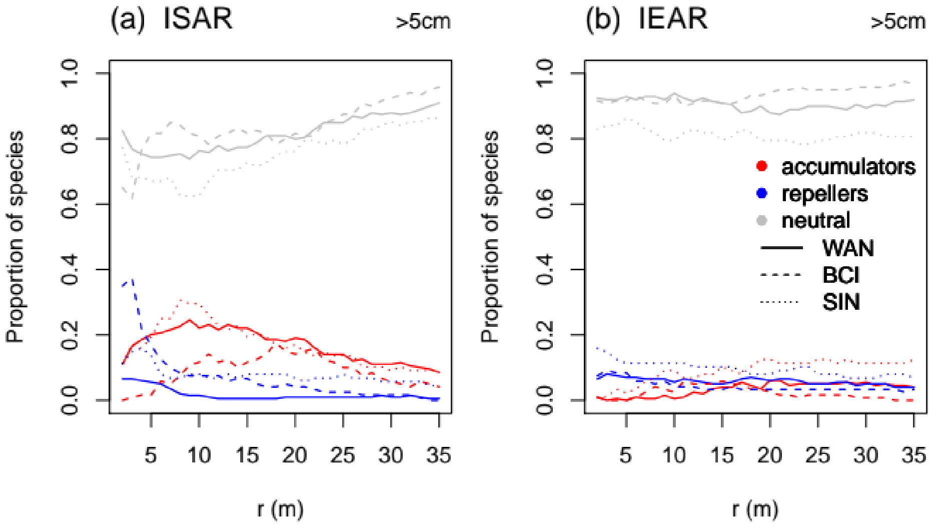

Fig. S3. Proportions of individual species showing more (i.e. accumulator), less (i.e. repeller) and expected (i.e. neutral) richness (ISAR) and evenness (IEAR) associations for trees with >5 cm DBH trees in WAN, BCI and SIN tropical forest plots with increasing spatial distance (radius r(m)). Species were classified by MAD test (Wiegand & Moloney 2014) where observed values were higher than global envelope limits of inhomogeneous null model for accumulators, lower for repellers or within the null model for neutral species.
