# Supplementary material for: Common spatial patterns of trees in various tropical forests: Small trees are associated with increased diversity at small spatial scales
Source: Ecol Evol. 2021 May 27;11(12):8085–95. doi: 10.1002/ece3.7640 (PMC8216943; doi:10.1002/ece3.7640)
Supplement: Supplementary file 2 — Supplementary Material [file ECE3-11-8085-s001.pdf]

**B. Supplementary materials of "Common spatial patterns of trees in various tropical forests: small trees are associated with increased diversity at small spatial scales" by Fibich P, Novotný V, Ediriweera S, Gunatilleke S, Gunatilleke N, Molem K, Weiblen GD, Lepš J.**

*Methods of individual species spatial patterns*

Diversity patterns are affected by the underlying spatial patterns of individual species, therefore the pair correlation function (PCF, g(r), also known as neighbourhood density function) defined as $g(r) = K'(r)/2\pi r$, where K'(r) is a derivation of Ripley's K function (Wiegand and Moloney 2014) was applied. The values of g(r) were compared with two null models. (1) The inhomogeneous Poisson null model (INM; described in the *Methods*) and (2) homogeneous Poisson null model (HNM, complete spatial randomness model), where the density is constant and independent of location within the plot (thus not affected by a possible environmental heterogeneity). Species were considered clumped (aggregated) or regular (segregated) at a given spatial scale (r) if the observed g(r) values were respectively above or below 95% null model simulation envelopes, and random if observed g(r) values were inside simulation envelopes. Pronounced differences between results based on HNM and INM null models suggest strong environmental gradient that species responds to (Wiegand and Moloney 2014). Ripley's isotropic edge correction was applied for g(r). Simulation envelopes definition, number of null model realizations and applied goodness-of-fit test were the same as for ISAR (see *Methods*).

*Results of individual species spatial patterns*

A peak in the proportion of clumped trees (25-40% of all species, depending on the plot) was observed at 5-10 m spatial scale for large trees (DBH>10 cm), when tested against INM (Fig. SB1a). The proportion of regularly distributed species was low, always <10% of species, at the scales from 0 to 30 m, then slightly increasing in WAN and SIN (the results above 35 m spatial scale reflects trees density gradient, because INM applies separation of scales approach). The trends were similar for trees with DBH > 5 cm, but less pronounced (Fig. SB1b). However, when all trees were analysed, the proportion of clumped species was high at 0-5 m scales (from 65% in SIN to 95% in WAN), then monotonically decreasing to values <10% at 25 m (SIN) or 50 m (WAN and BCI). The evidence for regularly distributed species was lacking at short spatial scales, increasing at larger scales to 10% (WAN, BCI) or even to 25% (SIN) (Fig. SB1c). The homogeneous

null model (HNM) showed higher proportions of clumped species than INM (Fig. SB2). The proportion of clumped species was the highest in SIN, lower in BCI and the lowest in WAN, except for the analysis of all trees where all plots had a similar proportion of clumped species. There were virtually no regularly dispersed species at any scale in the HNM, and the decrease in clumped species with increasing scale was also much slower than in the INM.

*References*

Wiegand, T. and Moloney, A.K. 2014. Handbook of spatial point-pattern analysis in ecology. – Chapman & Hall/CRC Press, Boca Raton, FL, US.

*Figures*

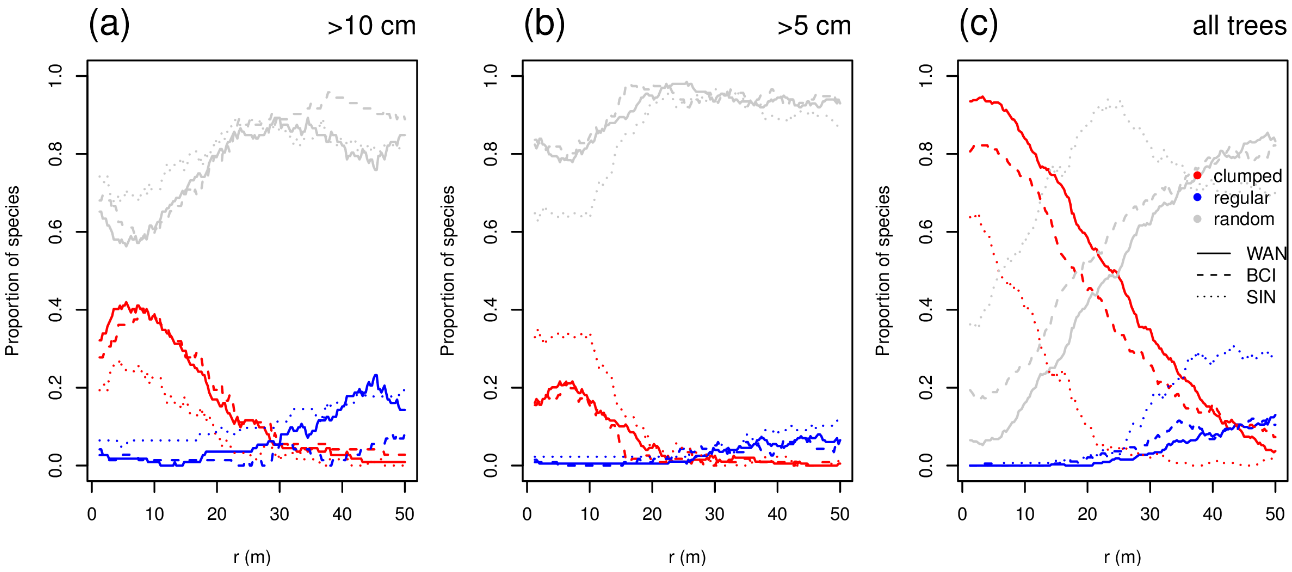


**Fig. SB1.** Proportions of individual species spatial patterns in WAN, BCI and SIN tropical forest plots for trees with DBH >10 cm, >5 cm and all trees (≥1 cm) with increasing spatial scale (radius r(m)). Clumped species, regular and random species are classified according a pair correlation function (g(r)) under inhomogeneous null model (having observed values higher, lower or inside 95 % null model simulation envelopes, respectively).

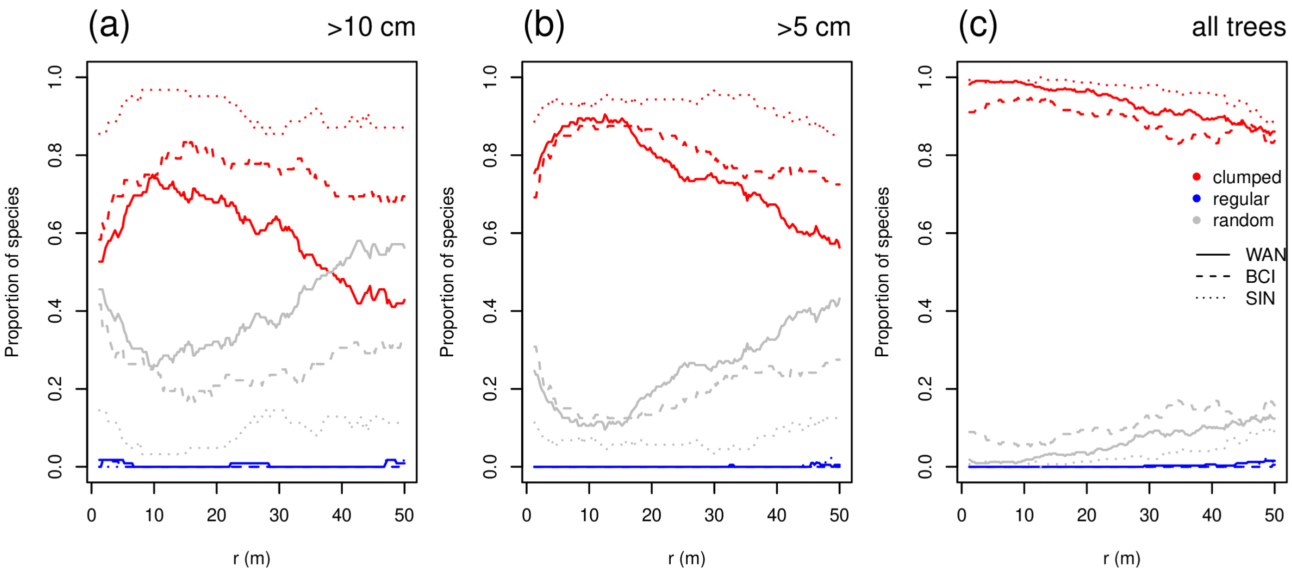


**Fig. SB2** Proportions of individual species spatial patterns in WAN, BCI and SIN tropical forest plots for trees with DBH > 10 cm, > 5 cm and all trees (≥1 cm) with increasing spatial scale (radius r(m)). Clumped species, regular and random species are classified according a pair correlation function (g(r)) under homogeneous null model (having observed values higher, lower or inside 95% null model simulation envelopes, respectively).
